# The *Ih* Channel Gene Promotes Synaptic Transmission and Coordinated Movement in *Drosophila melanogaster*

**DOI:** 10.3389/fnmol.2017.00041

**Published:** 2017-02-24

**Authors:** Andrew P. Hegle, C. Andrew Frank, Anthony Berndt, Markus Klose, Douglas W. Allan, Eric A. Accili

**Affiliations:** ^1^Department of Cellular and Physiological Sciences, The University of British ColumbiaVancouver, BC, Canada; ^2^Department of Anatomy and Cell Biology, The University of IowaIowa City, IA, USA

**Keywords:** *Ih* gene, HCN channel, neuromuscular junction, presynaptic mechanisms, *Drosophila melanogaster*

## Abstract

Hyperpolarization-activated cyclic nucleotide-gated “HCN” channels, which underlie the hyperpolarization-activated current (I_h_), have been proposed to play diverse roles in neurons. The presynaptic HCN channel is thought to both promote and inhibit neurotransmitter release from synapses, depending upon its interactions with other presynaptic ion channels. In larvae of *Drosophila melanogaster*, inhibition of the presynaptic HCN channel by the drug ZD7288 reduces the enhancement of neurotransmitter release at motor terminals by serotonin but this drug has no effect on basal neurotransmitter release, implying that the channel does not contribute to firing under basal conditions. Here, we show that genetic disruption of the sole HCN gene *(Ih)* reduces the amplitude of the evoked response at the neuromuscular junction (NMJ) of third instar larvae by decreasing the number of released vesicles. The anatomy of the (NMJ) is not notably affected by disruption of the *Ih* gene. We propose that the presynaptic HCN channel is active under basal conditions and promotes neurotransmission at larval motor terminals. Finally, we demonstrate that *Ih* partial loss-of-function mutant adult flies have impaired locomotion, and, thus, we hypothesize that the presynaptic HCN channel at the (NMJ) may contribute to coordinated movement.

## Introduction

Hyperpolarization-activated Cyclic Nucleotide-gated (HCN) channels underlie the hyperpolarization-activated current, designated I_h_ (hyperpolarization-activated current) or I_f_ (funny current). HCN channels have a primary structure that is similar to voltage-gated potassium channels (Gauss et al., 1998; Ludwig et al., 1998; Santoro et al., 1998); they are tetrameric and contain individual subunits which are multi-membrane spanning and possess intracellular N- and C-termini (Lee and Mackinnon, 2017). There are four HCN genes in mammals (HCN1-HCN4), which are expressed at high level in the heart and nervous system (Accili et al., 2002; Robinson and Siegelbaum, 2003; Biel et al., 2009). HCN channels are activated by membrane hyperpolarization, and their opening is facilitated by direct binding of cAMP to a cyclic nucleotide binding site in the C-terminus (Difrancesco, 1981a; Difrancesco and Tortora, 1991; Wainger et al., 2001; Zagotta et al., 2003; Chow et al., 2012); the rate of opening and closing, and the effect of cAMP, varies between the isoforms, which are coded by different genes (Altomare et al., 2001; Robinson and Siegelbaum, 2003). In the mammalian heart, HCN channels are especially enriched in the sinoatrial node, where their unique activation properties and sensitivity to cAMP have been proposed to promote pacemaker activity and proper electrical conduction (Difrancesco, 1993; Baruscotti et al., 2011). HCN channels and the currents they produce have also been identified in a wide range of vertebrate and invertebrate neurons where they have varying patterns of cellular localization and are thought to contribute to presynaptic and postsynaptic functions (Pape, 1996; Robinson and Siegelbaum, 2003; Biel et al., 2009).

Hyperpolarization-activated Cyclic Nucleotide-gated (HCN) channels have mixed cation permeability, they open and close with time constants that range from hundreds of milliseconds to seconds, depending on membrane voltage and the isoform involved, and mammalian isoforms do not inactivate (Difrancesco, 1981b; Pape, 1996; Robinson and Siegelbaum, 2003; Biel et al., 2009). The opening of HCN channels by hyperpolarization leads to depolarization of the resting membrane potential to ~−25 mV, which is the reversal potential for the channels in physiological solutions. Curiously, presynaptic HCN channels have been proposed to both promote and inhibit neurotransmitter release (Biel et al., 2009; Tomlinson et al., 2010; Trevillion et al., 2010; Howells et al., 2012; Huang and Trussell, 2014; Lorenz and Jones, 2014; Shah, 2014), which may depend upon the context provided by the complement of other ion channels present in particular axons or presynaptic terminals. For example, in mice, genetic deletion of the HCN1 gene or pharmacological inhibition of I_h_ by the drug ZD7288 have been shown to enhance the frequency of miniature excitatory post-synaptic potentials onto EC layer III pyramids by promoting the entry of calcium through T-type calcium channels; this suggests that I_h_ normally limits calcium entry into the synapse and reduces neurotransmission (Huang et al., 2011). Using a combination of genetic approaches, the presynaptic HCN channel has also been shown to reduce glutamate release from amacrine cells in flies by limiting calcium channel activity (Hu et al., 2015). Conversely, pharmacological inhibition of the presynaptic HCN channel by ZD7288 has been shown to reduce the stimulation of neurotransmitter release by serotonin at the neuromuscular junction (NMJ) of *Drosophila melanogaster* larvae and crayfish; notably, however, ZD7288 has no apparent effect on basal release (Beaumont and Zucker, 2000; Cheung et al., 2006).

Based on these pharmacological data in *D. melanogaster*, it appears that I_h_ does not impact basal neurotransmission. However, many of the studies that examine the presynaptic HCN channel use I_h_ inhibitors which are not specific. Perhaps the most commonly used I_h_ inhibitor is ZD7288, which requires a relatively long incubation and, when used at lower concentrations (<1 μM), does not inhibit I_h_ completely (Bosmith et al., 1993; Qu et al., 2008). Thus, ZD7288 is often used at concentrations which are known to affect other ion channels.

Few studies have exploited genetic strategies to either corroborate the actions of ZD7288 on neuronal HCN channels and I_h_ (Huang et al., 2011) or to avoid its use altogether (Hu et al., 2015). To this end, we examined the function of the single HCN gene in *D. melanogaster, Ih*. We found that *Ih* null embryos are unable to hatch, whereas *Ih* hypomorphs are viable but as adults exhibit long periods of inactivity, impaired climbing and decreased lifespan. To obtain direct evidence for a contribution of *Ih* to presynaptic signaling, we performed electrophysiological recordings at the (NMJ) of late third instar larvae. We found that the evoked potentials and quantal content were reduced in *Ih* hypomorphs. We propose that presynaptic I_h_ promotes basal neurotransmitter release at the presynaptic motor terminal as well as serotonin-induced neurotransmitter release which was shown previously (Beaumont and Zucker, 2000; Cheung et al., 2006). Finally, we found that *Ih* hypomorphic adult flies display a notable lack of coordinated movement. Promotion of neurotransmission at motor terminals by the presynaptic HCN channel may explain the movement phenotypes of *Ih* mutant flies.

## Methods

### Genetics

**Figure 1:**
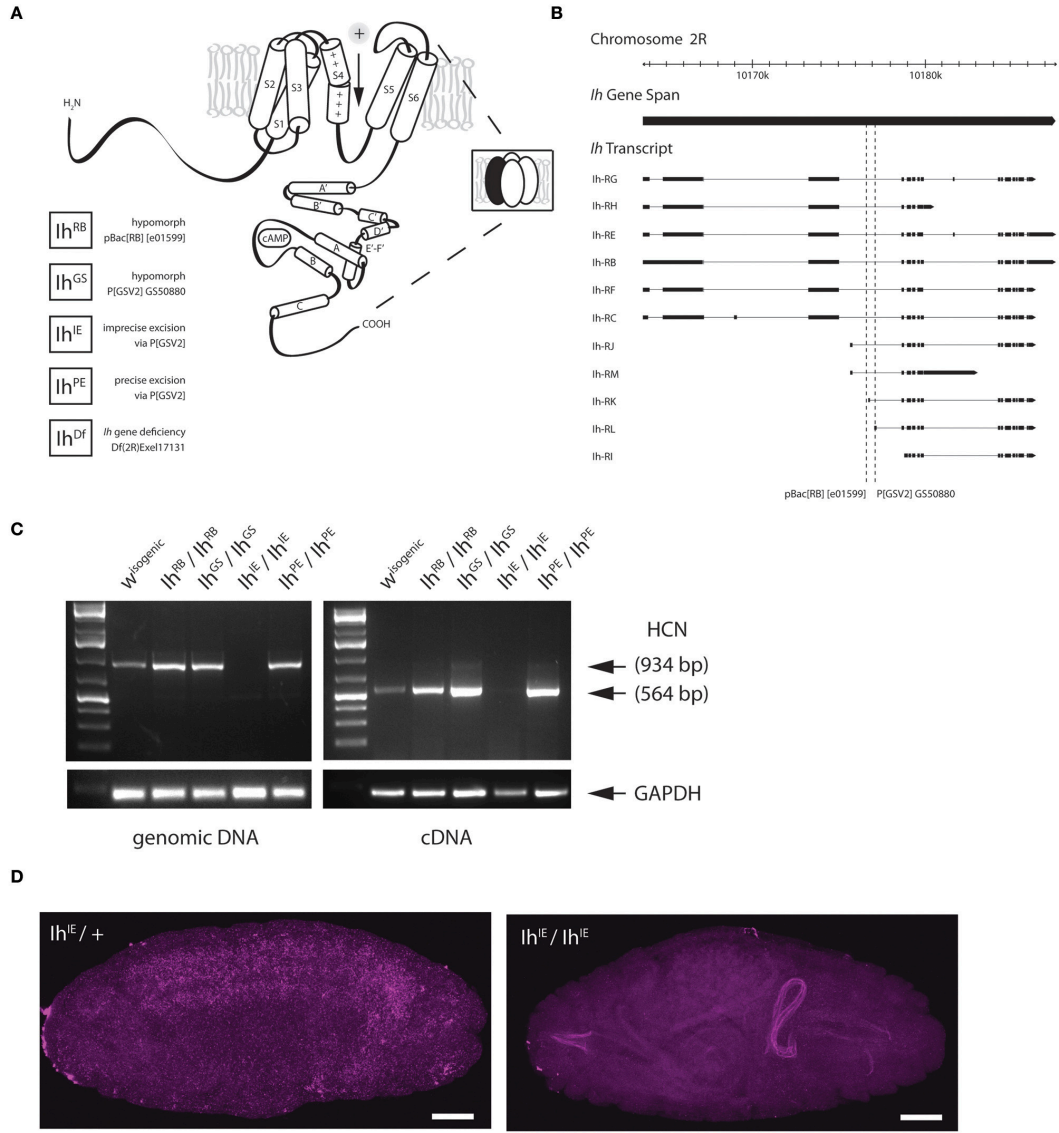
**Genetic excision of the fly HCN channel. (A)** A cartoon showing the predicted topology of a full-length HCN channel subunit in *Drosophila melanogaster*. Functional channels consist of four subunits (inset) and open in response to membrane hyperpolarization. All *Ih* alleles used in this study are listed on the lower left. The GS transposable element (Flybase P[GSV2] GS50880) was used to generate precise and imprecise excisions. Cartoon adapted from Biel et al. (2009). **(B)** A genomic map of the *Ih* locus on the second chromosome showing exon structure of *Ih* transcripts. The locations of transposable element insertions are intronic and are indicated with dotted lines. Map adapted from Flybase. **(C)** A gel showing fragments produced by genomic PCR (left) and reverse-transcriptase PCR (right), after genetic disruption of *Ih* in E17 embryos as indicated. All lines were out-crossed five times to an isogenic *w-* wild type line. Primers correspond to the region of exons 4–6 in *Ih* mRNA, immediately downstream of the intronic *P[GSV2]* insertion site. **(D)** Confocal images of embryos comparing *in situ* hybridization of *Ih* mRNA expression in *Ih*^*IE*^*/CyO,Twi-GFP* heterozygotes (left) with that of *Ih*^*IE*^*/Ih*^*IE*^ null embryos (right). Digoxigenin-UTP-labeled RNA probes to the *Ih* gene were generated using the same primers as in **(C)**. Note the absence of *Ih* expression in *Ih*^*IE*^*/Ih*^*IE*^ null embryos. Fluorescence on the right arises from autofluorescence of the larval gut. Scale bars = 50 μm.

The *P[GSV2] GS50880* transposable element insertion in the *Ih* gene (*CG8585*) was crossed to a Δ*2-3 transposase* line to generate a series of precise and imprecise genomic excision events. Candidate alleles were screened using PCR and complementation analysis with an *Ih* deficiency allele, *Df (2R)Exel[17131]*. A precise excision line with the smallest footprint (32 bp) was used as a control allele. Although three start codons remain in the excised *P[GSV2]* footprint, it was not predicted to disrupt any of the coding sequences of the gene, and *Ih* cDNA immediately downstream of the insertion site was not reduced in flies containing this footprint (Figure 1C). Importantly, precise excision *Ih*^*PE*^ alleles exhibited wild type gene activity in all molecular and behavioral tests. Imprecise excision events were observed in two lines, which did not produce homozygotic animals and failed to complement a deficiency through *Ih*. Reverse-transcriptase PCR was used to test transcript levels in different alleles; confirming transcript loss in imprecise excision *Ih*^*IE*^ (null) and wild type levels in precise excision *Ih*^*PE*^ (revertant control). Candidate alleles, including precise and imprecise alleles, as well as the original insertion line(s) were out-crossed five times to an isogenic *w*^−^ strain to eliminate potential second site mutations and improve their utility in behavioral assays. Mutants were kept over *CyO,Act-GFP TM3,Ser,Act-GFP* or *CyO, twiGAL4,UAS-2xEGFP* or *TM3,Sb,Ser,twiGAL4,UAS-2xEGFP*. Isogenic *w*^1118^ was used as the control genotype. Flies were maintained at 25°C, 70% humidity on standard cornmeal food.

### Molecular biology

Genomic DNA was extracted from Stage 17 embryos, late third instar larvae, or freshly-eclosed adult flies using DNAzol (Invitrogen) for screening through the entire *Ih* locus using PCR. For reverse-transcriptase PCR reactions, total RNA was extracted using Qiazol (Qiagen), followed by standard chloroform precipitation and purification with the Zymogen RNA clean-up kit. Total RNA was reverse transcribed with the iScript cDNA synthesis kit (BioRad). The following primers were utilized to determine the presence or absence of exons 4-6: 5′-GCTGCTCCTATTGCTCGGTG, 3′-GTTCAGCGTTGTCTTGTTGC. This region translates to the first three transmembrane domains of the wild type channel.

### *In situ* hybridization and imaging

Digoxigenin-UTP-labeled RNA probes to the *Ih* gene were generated using the Roche DIG labeling kit. Primers used were the same as described above. For each allele, timed grape plate collections were used to collect age-matched 12–15 h embryos, which were immediately fixed in 4% PBS-buffered formaldehyde. Probe hybridization was carried out as described in Weiszmann et al. (2009). Briefly, embryos were incubated overnight at 55°C in a solution containing DIG-labeled probe in 50% formamide, 3X saline-sodium citrate buffer and 0.01% Tween-20. Embryos were washed extensively, blocked with 5% donkey serum in 0.1% Triton-X-100 and incubated for 2 h with α-DIG-POD (Fab fragments from polyclonal anti-digoxigenin antibodies, conjugated to horseradish peroxidise used at a dilution of 1:2000), followed by 30 min developing with a tyramide signal amplification (TSA) developing agent. Embryos were then washed and incubated overnight with a chicken α-GFP 1° antibody (1:1000). The following day, embryos were incubated for 1 h with α-chicken-Alexa 649 2° antibody (1:500), washed and mounted on slides with VectaShield.

For imaging of the (NMJ) at muscles 4 and 6/7, third instar larval filets were pinned out on Sylgard and fixed with 4% PBS-buffered formaldehyde. Fixed filets were blocked for 2 h with 5% donkey serum in 0.1% Triton-X-100, followed by overnight incubation with mouse α-Bruchpilot (1:50). The following day, filets were washed and re-blocked for 1 h, followed by a 2 h incubation in the dark with α-mouse Cy3 (1:100), α-HRP Cy5 (1:50) and α-phalloidin FITC (1:500), washing and mounting with VectaShield. All imaging was performed using an Olympus FV1000 laser scanning confocal microscope and imaged processed in Adobe Photoshop.

### Behavior

For embryo hatching assays, timed embryo collections were used to collect age-matched Stage 17 embryos overnight. For each genotype, 20–50 dechorionated embryos were isolated on one side of a fresh grape agar plate with a small amount of yeast paste on the opposite side. After 6 h, the numbers of hatched and unhatched embryos were counted. Plates were re-examined after 24 h, and in all cases embryos that did not hatch in the first 6 h remained unhatched. A minimum of 100 embryos were examined for each genotype. The genotypes were compared using Fisher's exact test.

For climbing assays, age-matched, and freshly eclosed male adult flies from each genotype were collected in groups of ten. Each vial was assayed for climbing at days 1, 5, 9, 13, 17, and 21 post-eclosion. Climbing index was calculated as the average percentage of flies to reach a 4 cm line in 8 s after they were gently tapped to the bottom of a clean, empty vial. For each genotype, 4–6 sets of 10 males were measured four times and averaged, first among trials and then among sets. One-way ANOVA with Tukey's multiple comparisons test was performed among all genotypes for each day. For lifespan assays, fifty age-matched adults were isolated by sex in groups of ten. The number of dead flies was recorded each day until no live flies remained. Fresh food was provided daily.

### Electrophysiology

Sharp electrode recordings were taken from muscle 6 in abdominal segments 2 or 3 of third instar larvae, as previously described (Jan and Jan, 1976; Frank et al., 2006, 2009; Brusich et al., 2015). For recording, sharp electrodes were pulled using a P-97 micropette puller (Sutter Instruments) to an approximate resistance of 15 MΩ and filled with 3 M KCl. Larvae were dissected in a modified HL3 saline containing: NaCl (70 mM), KCl (5 mM), MgCl_2_ (10 mM), NaHCO_3_ (10 mM), sucrose (115 mM = 3.9%), trehalose (4.2 mM = 0.16%), HEPES (5.0 mM = 0.12%), and CaCl_2_ (0.5 mM). Only muscles with a resting potential of −60 mV or lower and an input resistance of 5 MΩ or greater were accepted for recording. Recordings were made using Axopatch 200B (in bridge mode) or Axoclamp 900A amplifiers (Molecular Devices, Sunnyvale, CA), digitized using a Digidata 1440A data acquisition system (Molecular Devices), and recorded with pCLAMP 10 acquisition software (Molecular Devices). For presynaptic nerve stimulation, a Master-8 pulse stimulator (A.M.P. Instruments, Jerusalem, Israel) and an ISO-Flex isolation unit (A.M.P. Instruments) were utilized to deliver 1 ms suprathreshold stimuli to the appropriate segmental nerve through a fire-polished glass electrode filled with recording saline. For evoked events, stimuli were applied at a frequency of 1 Hz. For evoked trains of stimuli (used to analyze paired-pulse responses), a frequency of 10 Hz was used.

For each NMJ, ~100–200 spontaneous miniature excitatory postsynaptic potential (mEPSP) events were recorded. Each spontaneous event was analyzed by hand. From this analysis, an average mEPSP size for each muscle was calculated, as was an average mEPSP frequency for each muscle. To generate mEPSP cumulative size distribution histograms for each genotype, the first 75–100 mEPSPs per muscle were chosen and added to the analysis. As a result, a minimum of 800 separate spontaneous events were analyzed per genotype.

Thirty evoked excitatory postsynaptic potentials (EPSP) per muscle were recorded and averaged. Quantal content (QC) was calculated for every muscle by dividing the average EPSP/average mEPSP. Since this EPSP/mEPSP QC estimate is accurate only for sufficiently small EPSPs, QC was then corrected for non-linear summation (NLS QC) as previously described (Martin, 1955). To correct QC for non-linear summation, the calculated EPSP/mEPSP quotient for each muscle was multiplied by a correction factor, derived from (Martin, 1955): (1–EPSP/V_o_)^−1^. V_o_ is defined as the maximum electromotive force and was determined for each NMJ based on the muscle resting potential.

Electrophysiological data for each genotype were reported as mean ± SEM, and analyzed for statistical significance using ANOVA with a Tukey's *post-hoc* test to account for multiple comparisons (GraphPad Prism). For the mEPSP cumulative probability histogram, data were analyzed for statistical significance using a Kruskal-Wallis test with a Dunn's multiple comparisons *post-hoc* test (GraphPad Prism).

## Results

### Imprecise excision of the single HCN gene, *Ih*, is homozygous lethal

The HCN gene in *D. melanogaster, Ih*, produces a channel that is similar in structure and function to the four mammalian HCN isoforms and other invertebrate HCN isoforms (Gauss et al., 1998; Ludwig et al., 1998; Santoro et al., 1998; Ishii et al., 1999; Marx et al., 1999; Gisselmann et al., 2005; Jackson et al., 2012). Despite their distinctive properties, HCN isoforms have significant sequence homology with channels in the voltage-gated potassium channel superfamily, for which the crystal structure has been solved (Doyle et al., 1998; Lee et al., 2005; Long et al., 2005). By extrapolation, HCN subunits are predicted to have six transmembrane helices (S1–S6) with a cytosolic N and C-terminus. The subunits combine as tetramers to form the ion conducting channel (Figure 1A). The C-terminus contains a cyclic nucleotide-binding domain, which is connected to the sixth transmembrane domain, which forms the pore, by a region known as the C-linker (Wainger et al., 2001; Robinson and Siegelbaum, 2003; Zagotta et al., 2003).

To disrupt the *Ih* gene in *Drosophila*, we used transposase-based excision of an intronic P element. In Figure 1B, the position of two intronic insertional mutants, the P element *P[GSV2]GS50880* and the piggyBac element *pBac(RB)[e01599]*, are shown relative to the *Ih* gene structure. These insertions were selected because they occur in an intron shared by most predicted *Ih* transcripts (Figure 1B). We mobilized the *P[GSV2]* element and screened a large set of independent excision lines for lethality, then used PCR to amplify regions of the *Ih* locus from the most viable and most lethal strains by PCR to identify the underlying molecular lesion. We isolated a viable precise excision strain, designated *Ih*^*PE*^, for use as a revertant control. Several homozygous lethal excision alleles also failed to complement an *Ih* deficiency, *Ih*^*Df*^. Of these, a lethal imprecise excision strain, designated *Ih*^*IE*^, had a large deletion that ablated the entire *Ih* locus. All alleles were outcrossed five times to an isogenic *w*^1118^ control strain in order to remove second-site mutations.

Next, we confirmed that *Ih* mRNA is absent in the *Ih*^*IE*^ genotype by reverse-transcriptase polymerase chain reaction (RT-PCR) and *in situ* hybridization. Figure 1C shows the amplification of both genomic (left) and complementary DNA (right) purified from stage 17 embryos. The amplified region corresponds to the first three transmembrane domains of the I_h_ channel, and was chosen for analysis as it is immediately downstream of the insertion site of the *P(GSV2)* transposable element. The band corresponding to this region is absent from *Ih*^*IE*^*/Ih*^*IE*^ embryonic gDNA and cDNA, but is present in *Ih*^*PE*^*/Ih*^*PE*^ controls. Notably, the band is also present in cDNA from *Ih*^*RB*^*/Ih*^*RB*^ and *Ih*^*GS*^*/Ih*^*GS*^ embryos, suggesting that mRNA is spliced normally in spite of the large transposon insertions immediately upstream.

To confirm mRNA knockdown and observe the wild type distribution of *Ih* message, *in situ* hybridization was also performed on *Ih*^*IE*^ embryos. In *Ih*^*IE*^*/CyO, Twi-GFP* heterozygotes, a DIG-labeled RNA probe to the same region as in Figure 1C generates a hybridization signal throughout the central nervous system (Figure 1D, left). By contrast, *Ih*^*IE*^ homozygotes exhibit a complete absence of neuronal DIG labeling (right), indicating that *Ih* mRNA is absent from these embryos, supporting our conclusion that *Ih*^*IE*^ is a null.

### *Ih* nulls are late embryonic lethal

**Figure 2:**
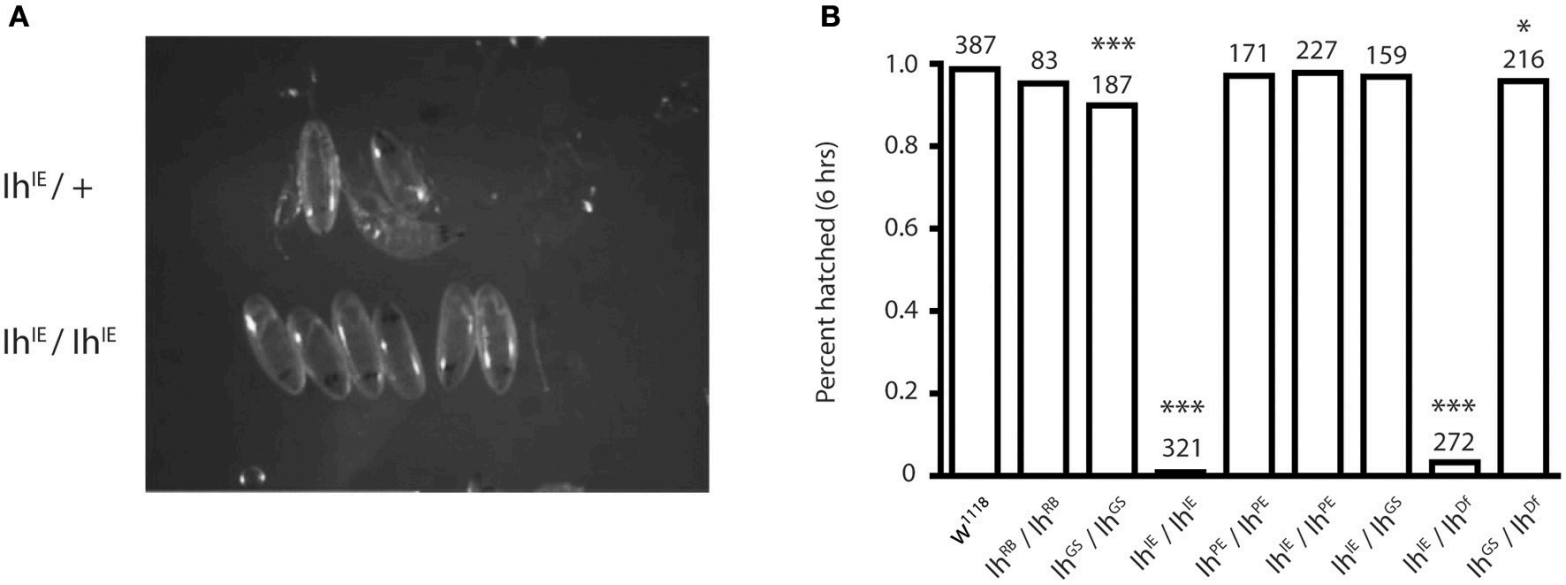
**Homozygotic excision of the ***Ih*** gene is embryonic lethal. (A)** Photograph comparing stage-matched heterozygous and homozygous *Ih*^*IE*^ embryos. *Ih*^*IE*^*/*+ embryos (upper row) hatch normally at stage 17, whereas *Ih*^*IE*^*/Ih*^*IE*^ embryos develop to stage 17 but fail to hatch and die within 24 h. **(B)** Bar graph showing the percentage of hatched embryos among an *Ih* allelic series. Stage-matched embryos were aligned on grape plates and hatching was scored after 6 and 24 h (not shown). *Ih*^*IE*^*/Ih*^*IE*^ and *Ih*^*IE*^*/Ih*^*Df*^ embryos had a near-zero hatching rate. Fisher's exact test was used to determine *p*-values ^*^p < 0.05, ^***^p < 0.0001.

To determine the *Ih*^*IE*^ null lethal phase, we isolated dechorionated *Ih*^*IE*^*/Ih*^*IE*^ embryos from their heterozygous siblings at embryonic stage 17 to observe hatching behavior. Homozygous embryos were observed for 24 h, and in all cases died without hatching (Figure 2A). When the vitelline membrane was removed manually, homozygotic larvae displayed severely reduced motility and did not develop beyond the first instar stage, dying within 24 h. This observation suggests that the *Ih* gene is essential and that channel may play a role in larval locomotion. Figure 2B shows that, within 6 h, the percentage of hatched eggs was severely reduced in the *Ih*^*IE*^*/Ih*^*IE*^ and *Ih*^*IE*^*/Ih*^*Df*^ genotypes and only mildly reduced in the *Ih*^*GS*^/*Ih*^*GS*^ and *Ih*^*GS*^/*Ih*^*Df*^ genotypes.

### Disruption of the *Ih* gene does not modify larval synaptic morphology but reduces the number of effective vesicles released by presynaptic terminals

**Figure 3:**
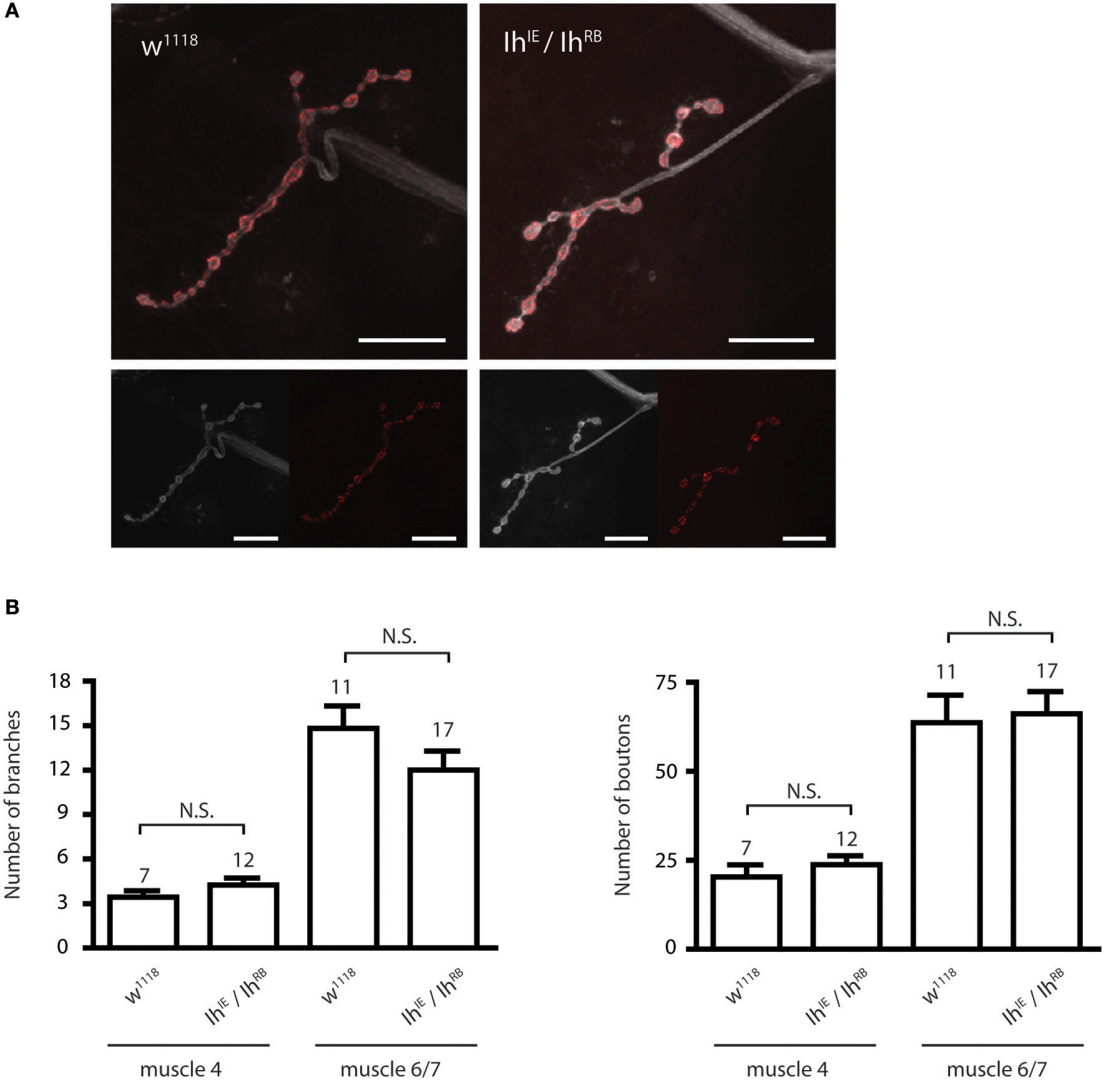
*****Ih*** disruption does not modify synaptic morphology in larval motor terminals. (A)** Confocal images of the muscle 4 neuromuscular junction in wild type (left) and *Ih*^*IE*^*/Ih*^*RB*^ (right) third instar larvae. Neuronal membranes were labeled with α-HRP (white) and synaptic active zones were labeled with α-Bruchpilot. Scale bars = 20 μm. **(B)** Histograms comparing the number of branches (left) and boutons (right) at the larval neuromuscular junction. No significant differences in branch or bouton number were observed between alleles (*p* > 0.05) using one-way ANOVA with Tukey's multiple comparisons test.

We next examined the morphology of the larval NMJ to determine if it was altered in third instar larvae with a disrupted *Ih* gene. For these experiments, we focused on non-lethal hypomorphic allelic combinations (i.e., *Ih*^*IE*^*/Ih*^*RB*^, *Ih*^*IE*^*/Ih*^*GS*^), which survive to the third instar larval stage and can thus be analyzed at late larval stages. Figure 3A shows representative confocal images of muscle 4 NMJs in third instar larval filets, stained with α*-*HRP to visualize neuronal membranes and α-Bruchpilot to reveal active zones. *Ih*^*IE*^*/Ih*^*RB*^ hypomorphic NMJs (right) were similar in appearance to those of wild type larvae (left), a result that was also observed at muscle 6/7 (not shown). Neuronal branching and number of boutons were quantified for both muscle 4 and muscle 6/7 NMJs, revealing no significant differences between the *Ih*^*IE*^*/Ih*^*RB*^ mutant NMJs and *w*^1118^ (isogenic wild-type) control NMJs (*p* > 0.05, Figure 3B).

**Figure 4:**
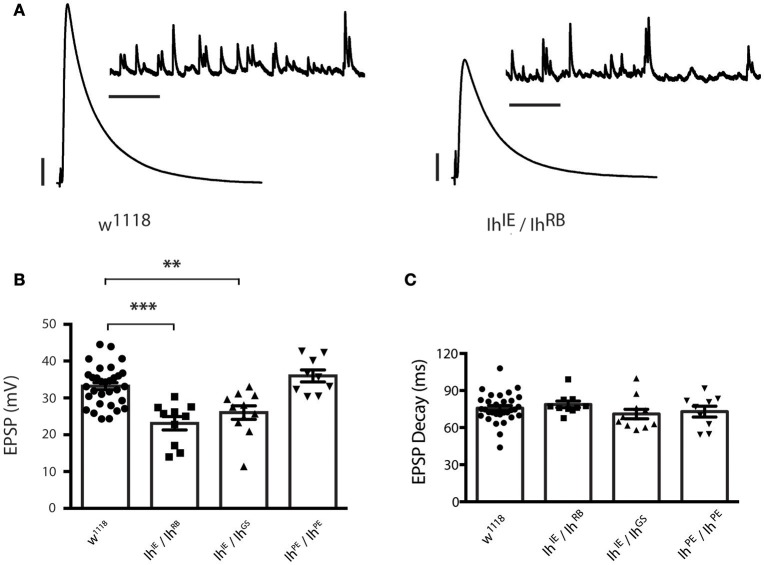
*****Ih*** disruption reduces evoked potentials in larval motor terminals. (A)** Representative electrophysiological recordings from the neuromuscular junction. Scale bars for EPSPs (mEPSPs): 5 mV (1 mV); 50 ms (1000 ms). **(B)** Plots of average evoked EPSPs in muscle recorded in response to nerve stimulation vs. genotype. Each point represents the average of 30 EPSPs recorded from a single muscle (1 Hz), and each bar represents the average (± SEM) of all muscles recorded for the given genotype. EPSPs recorded from both experimental groups (*Ih*^*IE*^*/Ih*^*RB*^ and *Ih*^*IE*^*/Ih*^*GS*^) are significantly smaller than those recorded from the wild type (^***^p < 0.001 for *Ih*^*IE*^*/Ih*^*RB*^ vs. *w*^1118^ control; ^**^p = 0.002 for *Ih*^*IE*^*/Ih*^*GS*^ vs. *w*^1118^ control by one-way ANOVA with Tukey's *post-hoc* to control for multiple group comparisons), whereas those recorded from the precise excision (*Ih*^*PE*^*/Ih*^*PE*^) are not significantly different from wild type EPSPs (*p* = 0.54 vs. *w*^1118^). **(C)** Plots of average EPSP decay (90% peak to 10% peak) times for all recorded genotypes. Point data and averages displayed as in 4B. No genotype showed any statistically significant difference in evoked potential decay (*p* ranged from 0.44 to 0.98 for all possible pairwise comparisons by one-way ANOVA with Tukey's *post-hoc*).

To determine the role of the *Ih* gene in neurotransmission at the NMJ, we carried out electrophysiological recordings from muscle 6/7 of third instar larvae (representative electrophysiological traces, Figure 4A). For the heteroallelic loss-of-function mutant combinations, *Ih*^*IE*^*/Ih*^*RB*^ and *Ih*^*IE*^*/Ih*^*GS*^, excitatory postsynaptic potentials (EPSPs) were significantly reduced compared to wild-type *w*^1118^ controls (Figure 4B). By contrast, larval NMJs homozygous for the precise excision *Ih*^*PE*^*/Ih*^*PE*^ allele had evoked potentials that were indistinguishable from wild type (Figure 4B). Together, these results suggest that loss-of-function lesions in the *Ih* locus impair neurotransmission. We noted that EPSP decay kinetics were unchanged between mutants and controls (Figure 4C). This result demonstrated unchanged timing for muscles to process a response to neurotransmitter, which likely reflects unchanged postsynaptic development.

**Figure 5:**
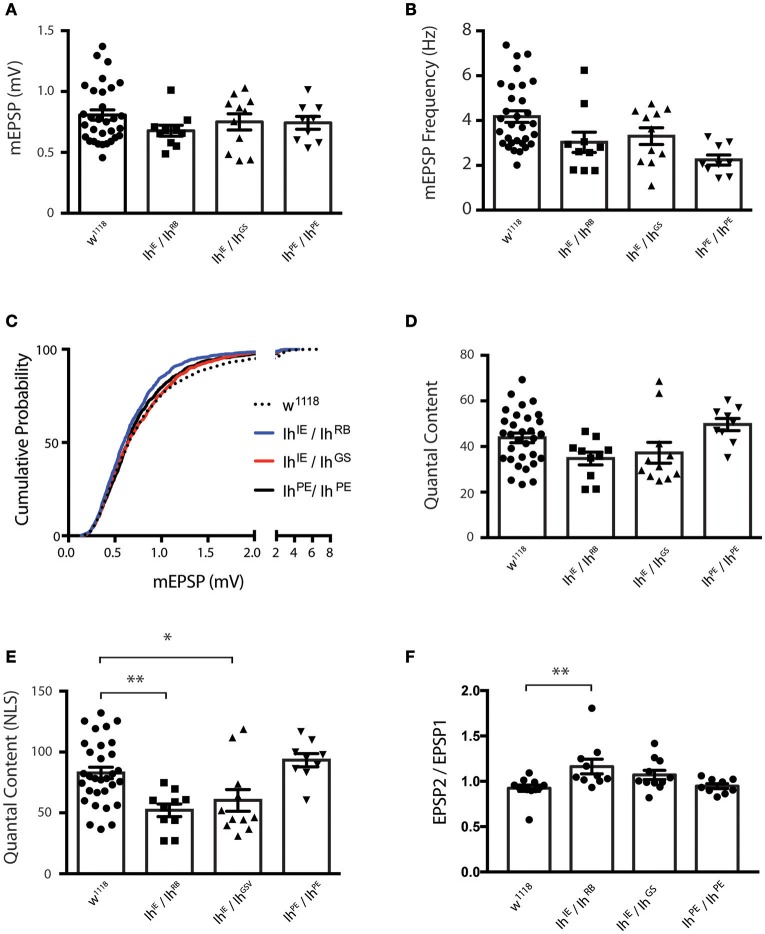
*****Ih*** disruption diminishes presynaptic vesicle release. (A)** Plots of average quantal size (mEPSP amplitude) for each genotype. Each point represents 100–200 spontaneous mEPSP events measured and averaged for a single muscle; each bar represents the average (± SEM) mEPSP amplitude for each genotype. There are no significant differences in average quantal size among the genotypes (*p* ranges from 0.35 to 0.99 for all possible pairwise comparisons by one-way ANOVA with Tukey's *post-hoc*). **(B)** Plots of average spontaneous mEPSP frequency for each genotype. Point data and averages displayed as described in 5A. Neither *Ih* hypomorphic combination significantly differs from controls (*p* > 0.1 vs. w1118 by one-way ANOVA with Tukey's *post-hoc*). **(C)** Cumulative probability histograms of quantal size for each genotype examined. A minimum of 800 spontaneous mEPSP events over a minimum of 9 NMJs were analyzed for each genotype. Compared to control genotypes, *Ih*^*IE*^*/Ih*^*RB*^ shows a slight diminishment in mEPSP size, assessed by cumulative probability (Kruskal-Wallis Test with Dunn's multiple comparisons *post-hoc*). However, *Ih*^*IE*^*/Ih*^*GS*^ shows no significant difference compared to controls. **(D)** Plots of quantal content (QC) determined from evoked potentials recorded in muscle in response to electrical stimulation vs. genotype. Each point represents the average of 30 EPSPs in one muscle divided by the average of 100–200 mEPSPs recorded from the same muscle. Bars are the average for each genotype (± SEM). Quantal content is reported as an uncorrected (average EPSP/average mEPSP). By this measure, both *Ih* hypomorphic combinations have diminished QC compared to *w*^1118^, but neither reaches statistical significance (*p* > 0.14 vs. *w*^1118^ by one-way ANOVA with Tukey's *post-hoc*). **(E)** Plots of quantal content corrected for non-linear summation (NLS, See Methods). Points and averages plotted as in 5D. Both Ih hypomorphic combinations have diminished NLS QC compared to w1118 (^**^p = 0.006 for *Ih*^*IE*^*/Ih*^*RB*^; ^*^p = 0.05 for *Ih*^*IE*^*/Ih*^*GS*^ vs *w*^1118^ one-way ANOVA with Tukey's *post-hoc*). **(F)** Paired-pulse plots (EPSP2/EPSP1) measured from short 10 Hz trains of evoked stimuli for each genotype. Point data are from the average of two separate paired pulse recordings per muscle and bars are the average (± SEM) for each genotype. The *Ih*^*IE*^*/Ih*^*RB*^ combination achieved statistical significance (^**^p = 0.01 by ANOVA vs. *w*^1118^). The *Ih*^*IE*^*/Ih*^*GSV*^ combination achieved a similar elevated EPSP2/EPSP1 ratio vs. *w*^1118^, but only trended toward statistical significance (*p* = 0.18).

Nevertheless, we were uncertain if the diminished evoked amplitudes for *Ih* mutants reflected diminished presynaptic neurotransmitter release or a diminished capacity of the muscle to detect neurotransmitter. To address this question, we turned to additional electrophysiological measures. We found that the average amplitude of spontaneous miniature excitatory postsynaptic potentials (mEPSPs)—an indicator of muscle sensitivity to single vesicles of neurotransmitter—was not significantly altered when comparing *Ih* mutants either to wild type or to precise excision controls (Figure 5A). Interestingly, mEPSP frequency varied somewhat between genotypes (Figure 5B), but was not significantly changed for the *Ih* mutants. Additionally, we measured and plotted the cumulative probability of mEPSP amplitude for all genotypes (minimum 800 mEPSPs analyzed per genotype, see Methods). By this analysis, there was a slight—and statistically significant—decrease in the cumulative size distribution of mEPSPs for the *Ih*^*IE*^*/Ih*^*RB*^ genetic combination (Figure 5C), but no significant difference between any of the other combinations.

To test the idea that diminished presynaptic vesicle release was responsible for reduced EPSP amplitudes, we calculated quantal content (QC) for each individual NMJ by dividing average EPSP amplitude/average mEPSP amplitude (Figure 5D). Since the evoked events were sufficiently large, we corrected QC for non-linear summation by using an established method (Martin, 1955; Figure 5E). We found a robust reduction in release for both *Ih* hypomorphic combinations, comparing either to wild-type controls or to precise excision *Ih*^*PE*^*/Ih*^*PE*^ controls (Figure 5E). This reduction is consistent with the reduction in EPSP amplitude. Our data suggest that reduced neurotransmission may account, at least in part, for the observed locomotor defects in homozygotic *Ih* mutant larvae.

To test the idea that probability of release could be decreased in *Ih* mutants, we challenged NMJs of all recorded genotypes with short 10 Hz stimulus trains and analyzed the amplitude of the first two pulses to determine the paired pulse ratio (EPSP2/EPSP1, Figure 5F). Classically, one would expect synapses with a lower probability of release to have higher paired pulse ratios–due to a rapid accumulation of Ca^2+^ in the presynaptic cleft and recruitment of presynaptic vesicles upon inducing a second pulse. Consistent with the EPSP data, both *Ih* mutant combinations showed a higher paired pulse ratio than wild-type and precise excision controls. For our data sets, only the *Ih*^*IE*^*/Ih*^*RB*^ combination achieved statistical significance (*p* = 0.01 by ANOVA vs. *w*^1118^). By contrast, the *Ih*^*IE*^*/Ih*^*GS*^ combination achieved a similar elevated EPSP2/EPSP1 ratio vs. *w*^1118^, but only trended toward statistical significance (*p* = 0.18).

### *Ih* hypomorphs exhibit adult motility defects

**Figure 6:**
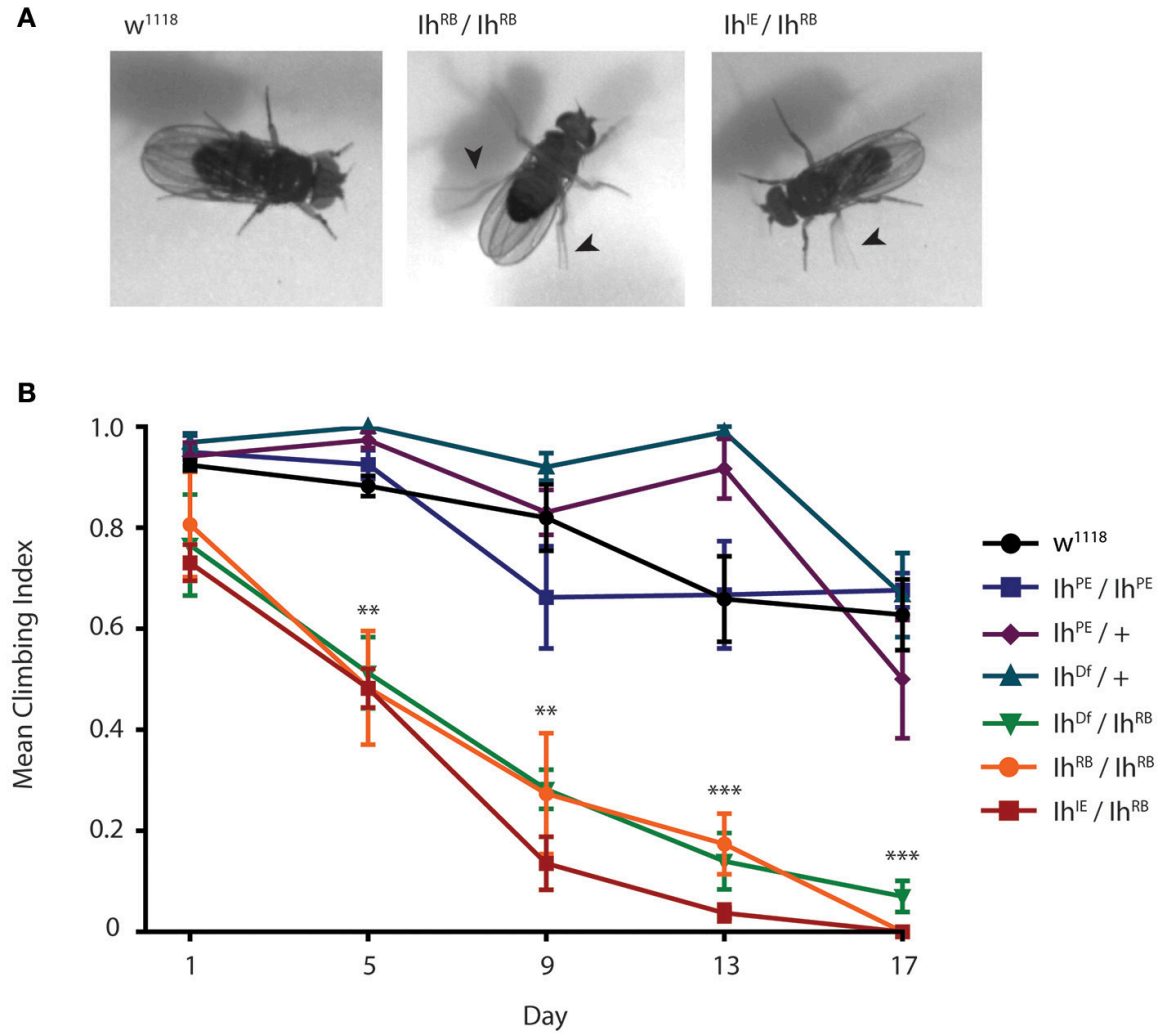
*****Ih*** disruption compromises adult motility in ***Drosophila melanogaster***. (A)** Photographs of wild type, *Ih*^*IE*^*/Ih*^*RB*^ and *Ih*^*RB*^*/Ih*^*RB*^ adult flies aged 15 days. Hypomorphic flies exhibit compromised posture and are hyper-excitable compared to wild type, including spontaneous leg tremors (arrowheads) and seizure-like episodes. **(B)** Graph of adult climbing ability over time for a series of *Ih* alleles, using a negative geotaxis assay. Groups of 8–10 flies were tested for their ability to climb 4 cm in 8 s. The percentage of flies reaching 4 cm (the climbing index) was averaged among 4 trials for each group. 4–6 vials per genotype were assessed every 5 days. Climbing index was observed to decrease significantly over time in *Ih* hypomorphic mutants compared to wild type using one-way ANOVA with Dunnet's multiple comparisons test. (^**^p < 0.001 by day 5, ^***^p < 0.0001 by day 17).

To examine how the *Ih* gene impacts motility in adult flies, we first examined flies containing transposon mutational insertions into the *Ih* gene. When we placed the two independent insertions, *P[GSV2]GS50880* and *pBac(RB)[e01599]*, over an *Ih* deficiency, *Ih*^*Df*^, we observed a similar motility phenotype in homozygous adult flies that included poor balance and spontaneous tremors as well as an abbreviated lifespan (Supplementary Figure 1). Due to the embryonic lethality of *Ih*^*IE*^*/Ih*^*IE*^ homozygotes, we focused on the *Ih*^*IE*^*/Ih*^*RB*^ genotype in order to characterize the effects of the *Ih* imprecise excision in adult flies. Figure 6A shows representative images of 15 day-old *Ih*^*IE*^*/Ih*^*RB*^ and *Ih*^*RB*^*/Ih*^*RB*^ adults, and highlights the hyperactive limb movement observed in these alleles compared to wild type adults. We found that the abnormal motility observed *Ih*^*IE*^*/Ih*^*RB*^ flies is comparable to that of *Ih*^*RB*^*/Ih*^*RB*^ flies (see Supplementary Videos 1–3).

In order to quantify the impaired motility of mutant *Ih* alleles, we assayed climbing behavior of adult flies (Figure 6B). A climbing index was established by measuring the percentage of adults able to climb four centimeters in 8 s. The climbing indices of *Ih*^*RB*^*/*+ and *Ih*^*Df*^*/*+ heterozygotes, as well as that of precise excision homozygotes (*Ih*^*PE*^*/Ih*^*PE*^) were not significantly different from wild type flies throughout the course of the experiment (*p* > 0.05 on day 17). In contrast, all hypomorphic allelic combinations tested, included *Ih*^*IE*^*/Ih*^*RB*^, *Ih*^*RB*^*/Ih*^*RB*^, and *Ih*^*Df*^*/Ih*^*RB*^, had a dramatically lower climbing index than that of wild type flies by day 5 after eclosion (Figure 6B, *p* < 0.001 in all comparisons). As predicted, the *Ih*^*IE*^*/Ih*^*RB*^ allele had the weakest climbing index, dropping to zero by the second week. Our finding that climbing ability is strongly compromised in a series of adult *Ih* mutant alleles is consistent with a role for the HCN channel in maintenance of basal synaptic function at the neuromuscular junction.

## Discussion

We examined the functional consequences of *Ih* gene disruption in *D. melanogaster*. Notably, electrophysiological recordings show that *Ih* gene hypomorphs exhibit reduced synaptic transmission at the NMJ in larvae because of a reduction in the number of effective vesicles released by presynaptic motor terminals. Previously, pharmacological inhibition of the presynaptic HCN channel by ZD7288 was shown to reduce the stimulation of neurotransmitter release by serotonin at the NMJ of *D. melanogaster* larvae, but this drug had no effect on basal neurotransmitter release in flies or in crayfish (Beaumont and Zucker, 2000; Cheung et al., 2006). A lack of an effect on basal release by ZD7288 could have resulted from a compensating inhibition of other ion channels or from an incomplete inhibition of I_h_ that still allows enough HCN channel activity to maintain basal release. Alternatively, the reduced evoked release we observed may be due to alterations in other presynaptic ion channels resulting from the chronic loss of the *Ih* gene.

How might loss of presynaptic HCN channels reduce the number of effective vesicles released? Hyperpolarization-induced activation of the HCN channel normally depolarizes the membrane potential because of its permeability to both sodium and potassium ions. Thus, the activity of presynaptic HCN channels may depolarize the membrane voltage in the distal axon and motor terminal, and stimulate calcium influx by activating voltage-gated calcium channels, which then promotes release of neurotransmitter-containing vesicles. Alternatively, the channel may interact directly with synaptic release machinery upon activation and promote neurotransmitter release; this has been suggested previously as the mechanism by which serotonin augments neurotransmitter release in the NMJ of the crayfish (Beaumont and Zucker, 2000).

Using a combination of genetic approaches, presynaptic I_h_ has been shown to reduce glutamate release from amacrine cells in flies by limiting the activity of Cacophony (Cac)-containing (Ca_V_2 alpha1 subunit) calcium channels (Hu et al., 2015). Ca_V_2 channels are critical for appropriate evoked neurotransmission at the NMJ, as well as the execution of plastic modulations of neurotransmission, as seen with homeostatic synaptic plasticity (Rieckhof et al., 2003; Xing et al., 2005; Frank et al., 2006; Frank, 2014). Based on the data garnered in amacrine cells, I_h_ could promote neurotransmission through Ca_*V*_2 regulation at the NMJ.

In the mouse, inhibition of low threshold voltage-gated T-type (Ca_V_3.2) Ca^2+^ channels by presynaptic I_h_ has also been noted in synaptic terminals in entorhinal cortical layer III pyramidal neurons (Huang et al., 2011). Thus, in general, the presynaptic HCN channel may either stimulate or inhibit neurotransmitter release, depending upon the neuronal type and the context provided by other ion channels in the terminal axon and synapse.

Disruption of the *Ih* gene leads to a range of behavioral defects in adult flies, which include tremors, imbalance, decreased motility, and shortened lifespan. Homozygotic disruption of the *Ih* gene by imprecise excision produced a lethal phenotype, in which embryos did not hatch. Even when the vitelline membrane was removed manually, null *Ih*^*IE*^ homozygotic larvae moved slowly and did not survive beyond the first instar (within 24 h). Thus, the inability of the embryos to hatch was likely due to reduced motor activity. A previous study carried out a partial *Ih* deletion strategy which produced an allele that did not cause embryonic lethality (Gonzalo-Gomez et al., 2012). The more robust phenotype observed in our null allele may be due to the fact that our approach using imprecise P-element excision ablated the majority of the *Ih* locus.

Adult hypomorphic mutant flies demonstrated an overall phenotype that is remarkably similar to that observed in mice either lacking the HCN2 isoform (Ludwig et al., 2003), or possessing a C-terminal truncated form of this isoform (Chung et al., 2009). Upon *HCN2* knockout, mice are hypoactive, show a wide-based and abnormal gait, and exhibit a tremor (Ludwig et al., 2003). Video recordings and EEGs show that these mice suffer from absence epilepsy and frequent concomitant spike-wave discharges, which contribute to hypoactivity. Mice possessing a C-terminal truncated HCN2 channel possess a phenotype which is referred to as *apathetic* (Chung et al., 2009); they exhibit ataxia, generalized spike–wave absence seizures, and tonic–clonic seizures. Homozygotic *apathetic* mice cannot coordinate swimming when forced to do so, and have difficulty with balance. For example, they fall to one side when standing on their hind legs to feed. Furthermore, apathetic homozygotes, but not heterozygotes, exhibit uncoordinated movement, unsteady gait and intention tremor. Finally, *apathetic* homozygotes die prematurely from unknown causes. The mechanism by which the genetic disruption of the I_h_/HCN channel leads to these phenotypes in mice remains unknown, but it could be related to its role in altering neurotransmission at the neuromuscular junction.

There are few published reports on the HCN channel in *D. melanogaster*. Disruption of the *Ih* gene has been proposed previously to decrease life span and modify sleep and dopamine levels in adult flies (Chen and Wang, 2012; Gonzalo-Gomez et al., 2012). It seems reasonable to expect that the fly model will yield significant insight into structure, function and physiology of mammalian HCN channels, given the pertinent data contained in the few studies carried out to date (Marx et al., 1999; Gisselmann et al., 2005; Cheung et al., 2006; Gonzalo-Gomez et al., 2012; Hu et al., 2015) and the considerable impact that the fly has had on other understanding closely-related voltage-gated ion channels (Littleton and Ganetzky, 2000; Cirelli et al., 2005). In particular, the fly offers a desirable model system to examine presynaptic I_h_ and its interactions with other presynaptic channels and proteins by exploiting genetic approaches and not relying exclusively on I_h_ inhibitor drugs, such as ZD7288.

## Ethics statement

This study was approved by the Office of Research Services at the University of British Columbia and the Office of The Institutional Animal Care and Use Committee at the University of Iowa.

## Author contributions

AH, DA, and EA conceived the original idea and experiments. AH, CAF, MK, AB, DA, and EA designed the experiments, and contributed to the analysis and interpretation of data. AH, CAF, DA, and EA edited the manuscript. AH organized and prepared the first draft of the paper, prepared the figures and, with EA, guided the editing process and prepared the final manuscript. AH, CAF, AB, and DA carried out the experiments.

## Funding

Our studies were funded by operating grants from the Canadian Institutes of Health Research to EA and DA and a Discovery Grant from the Natural Sciences and Engineering Research Council of Canada to EA. For this study, CAF was supported in part by NIH grant NS062738 and funds from the Department of Anatomy and Cell Biology, University of Iowa.

### Conflict of interest statement

The authors declare that the research was conducted in the absence of any commercial or financial relationships that could be construed as a potential conflict of interest.

## Supplementary material

Supplementary Figure 1 *****Ih*** disruption reduces viability of ***Drosophila melanogaster*** adults**. Graph showing the number of flies surviving over time for three genotypes. Flies with a P-element insertion in one copy (*Ih*^*RB*^*/*+) have a normal lifespan of ~60 days, as do flies lacking the HCN locus on one chromosome (*Ih*^*Df*^*/*+). In contrast, flies with disruptions in both copies of the HCN gene (*Ih*^*Df*^*/Ih*^*RB*^) have a significantly shortened lifespan (~20-day median). Starting populations of 50 flies per genotype were followed daily until all had died.

Supplementary Videos 1–3 **Climbing ability in 15 day-old ***Ih***^***IE***^/***Ih***^***RB***^ flies (video 2) and ***Ih***^***RB***^/***Ih***^***RB***^ flies (video 3) is comparable and both are compromised as compared to wild type control flies**.

## References

[B1] Accili E. A. Proenza C. Baruscotti M. Difrancesco D. (2002). From funny current to HCN channels: 20 years of excitation. News Physiol. Sci. 17, 32–37. 11821534 10.1152/physiologyonline.2002.17.1.32

[B2] Altomare C. Bucchi A. Camatini E. Baruscotti M. Viscomi C. Moroni A.. (2001). Integrated allosteric model of voltage gating of HCN channels. J. Gen. Physiol. 117, 519–532. 10.1085/jgp.117.6.519 11382803 PMC2232403

[B3] Baruscotti M. Bucchi A. Viscomi C. Mandelli G. Consalez G. Gnecchi-Rusconi T.. (2011). Deep bradycardia and heart block caused by inducible cardiac-specific knockout of the pacemaker channel gene Hcn4. Proc. Natl. Acad. Sci. U.S.A. 108, 1705–1710. 10.1073/pnas.1010122108 21220308 PMC3029742

[B4] Beaumont V. Zucker R. S. (2000). Enhancement of synaptic transmission by cyclic AMP modulation of presynaptic Ih channels. Nat. Neurosci. 3, 133–141. 10.1038/72072 10649568

[B5] Biel M. Wahl-Schott C. Michalakis S. Zong X. (2009). Hyperpolarization-activated cation channels: from genes to function. Physiol. Rev. 89, 847–885. 10.1152/physrev.00029.2008 19584315

[B6] Bosmith R. E. Briggs I. Sturgess N. C. (1993). Inhibitory actions of ZENECA ZD7288 on whole-cell hyperpolarization activated inward current (If) in guinea-pig dissociated sinoatrial node cells. Br. J. Pharmacol. 110, 343–349. 10.1111/j.1476-5381.1993.tb13815.x 7693281 PMC2176028

[B7] Brusich D. J. Spring A. M. Frank C. A. (2015). A single-cross, RNA interference-based genetic tool for examining the long-term maintenance of homeostatic plasticity. Front. Cell. Neurosci. 9:107. 10.3389/fncel.2015.00107 25859184 PMC4374470

[B8] Chen Z. Wang Z. (2012). Functional study of hyperpolarization activated channel (*Ih*) in *Drosophila* behavior. Sci. China Life Sci. 55, 2–7. 10.1007/s11427-012-4270-6 22314484

[B9] Cheung U. Atwood H. L. Zucker R. S. (2006). Presynaptic effectors contributing to cAMP-induced synaptic potentiation in *Drosophila*. J. Neurobiol. 66, 273–280. 10.1002/neu.20218 16329127

[B10] Chow S. S. Van Petegem F. Accili E. A. (2012). Energetics of cyclic AMP binding to HCN channel C terminus reveal negative cooperativity. J. Biol. Chem. 287, 600–606. 10.1074/jbc.M111.269563 22084239 PMC3249114

[B11] Chung W. K. Shin M. Jaramillo T. C. Leibel R. L. Leduc C. A. Fischer S. G.. (2009). Absence epilepsy in apathetic, a spontaneous mutant mouse lacking the h channel subunit, HCN2. Neurobiol. Dis. 33, 499–508. 10.1016/j.nbd.2008.12.004 19150498 PMC2643333

[B12] Cirelli C. Bushey D. Hill S. Huber R. Kreber R. Ganetzky B.. (2005). Reduced sleep in *Drosophila* Shaker mutants. Nature 434, 1087–1092. 10.1038/nature03486 15858564

[B13] Difrancesco D. (1981a). A new interpretation of the pace-maker current in calf Purkinje fibres. J. Physiol. 314, 359–376. 10.1113/jphysiol.1981.sp013713 6273533 PMC1249439

[B14] Difrancesco D. (1981b). A study of the ionic nature of the pace-maker current in calf Purkinje fibres. J. Physiol. 314, 377–393. 10.1113/jphysiol.1981.sp013714 6273534 PMC1249440

[B15] Difrancesco D. (1993). Pacemaker mechanisms in cardiac tissue. Annu. Rev. Physiol. 55, 455–472. 10.1146/annurev.ph.55.030193.002323 7682045

[B16] Difrancesco D. Tortora P. (1991). Direct activation of cardiac pacemaker channels by intracellular cyclic AMP. Nature 351, 145–147. 10.1038/351145a0 1709448

[B17] Doyle D. A. Morais Cabral J. Pfuetzner R. A. Kuo A. Gulbis J. M. Cohen S. L.. (1998). The structure of the potassium channel: molecular basis of K^+^ conduction and selectivity. Science 280, 69–77. 10.1126/science.280.5360.69 9525859

[B18] Frank C. A. (2014). How voltage-gated calcium channels gate forms of homeostatic synaptic plasticity. Front. Cell. Neurosci. 8:40. 10.3389/fncel.2014.00040 24592212 PMC3924756

[B19] Frank C. A. Kennedy M. J. Goold C. P. Marek K. W. Davis G. W. (2006). Mechanisms underlying the rapid induction and sustained expression of synaptic homeostasis. Neuron 52, 663–677. 10.1016/j.neuron.2006.09.029 17114050 PMC2673733

[B20] Frank C. A. Pielage J. Davis G. W. (2009). A presynaptic homeostatic signaling system composed of the Eph receptor, ephexin, Cdc42, and CaV2.1 calcium channels. Neuron 61, 556–569. 10.1016/j.neuron.2008.12.028 19249276 PMC2699049

[B21] Gauss R. Seifert R. Kaupp U. B. (1998). Molecular identification of a hyperpolarization-activated channel in sea urchin sperm. Nature 393, 583–587. 10.1038/31248 9634235

[B22] Gisselmann G. Gamerschlag B. Sonnenfeld R. Marx T. Neuhaus E. M. Wetzel C. H.. (2005). Variants of the *Drosophila melanogaster* Ih-channel are generated by different splicing. Insect Biochem. Mol. Biol. 35, 505–514. 10.1016/j.ibmb.2005.02.001 15804582

[B23] Gonzalo-Gomez A. Turiegano E. León Y. Molina I. Torroja L. Canal I. (2012). Ih current is necessary to maintain normal dopamine fluctuations and sleep consolidation in *Drosophila*. PLoS ONE 7:e36477. 10.1371/journal.pone.0036477 22574167 PMC3344876

[B24] Howells J. Trevillion L. Bostock H. Burke D. (2012). The voltage dependence of I(h) in human myelinated axons. J. Physiol. 590, 1625–1640. 10.1113/jphysiol.2011.225573 22310314 PMC3413487

[B25] Hu W. Wang T. Wang X. Han J. (2015). *Ih* channels control feedback regulation from amacrine cells to photoreceptors. PLoS Biol. 13:e1002115. 10.1371/journal.pbio.1002115 25831426 PMC4382183

[B26] Huang H. Trussell L. O. (2014). Presynaptic HCN channels regulate vesicular glutamate transport. Neuron 84, 340–346. 10.1016/j.neuron.2014.08.046 25263752 PMC4254032

[B27] Huang Z. Lujan R. Kadurin I. Uebele V. N. Renger J. J. Dolphin A. C.. (2011). Presynaptic HCN1 channels regulate Cav3.2 activity and neurotransmission at select cortical synapses. Nat. Neurosci. 14, 478–486. 10.1038/nn.2757 21358644 PMC3068302

[B28] Ishii T. M. Takano M. Xie L. H. Noma A. Ohmori H. (1999). Molecular characterization of the hyperpolarization-activated cation channel in rabbit heart sinoatrial node. J. Biol. Chem. 274, 12835–12839. 10.1074/jbc.274.18.12835 10212270

[B29] Jackson H. A. Hegle A. Nazzari H. Jegla T. Accili E. A. (2012). Asymmetric divergence in structure and function of HCN channel duplicates in Ciona intestinalis. PLoS ONE 7:e47590. 10.1371/journal.pone.0047590 23133599 PMC3487815

[B30] Jan L. Y. Jan Y. N. (1976). Properties of the larval neuromuscular junction in *Drosophila melanogaster*. J. Physiol. 262, 189–214. 10.1113/jphysiol.1976.sp011592 11339 PMC1307637

[B31] Lee C. H. Mackinnon R. (2017). Structures of the Human HCN1 Hyperpolarization-Activated Channel. Cell 168, 111–120.e111. 10.1016/j.cell.2016.12.023 28086084 PMC5496774

[B32] Lee S. Y. Lee A. Chen J. Mackinnon R. (2005). Structure of the KvAP voltage-dependent K+ channel and its dependence on the lipid membrane. Proc. Natl. Acad. Sci. U.S.A. 102, 15441–15446. 10.1073/pnas.0507651102 16223877 PMC1253646

[B33] Littleton J. T. Ganetzky B. (2000). Ion channels and synaptic organization: analysis of the *Drosophila* genome. Neuron 26, 35–43. 10.1016/S0896-6273(00)81135-6 10798390

[B34] Long S. B. Campbell E. B. Mackinnon R. (2005). Crystal structure of a mammalian voltage-dependent Shaker family K^+^ channel. Science 309, 897–903. 10.1126/science.1116269 16002581

[B35] Lorenz C. Jones K. E. (2014). IH activity is increased in populations of slow versus fast motor axons of the rat. Front. Hum. Neurosci. 8:766. 10.3389/fnhum.2014.00766 25309406 PMC4174588

[B36] Ludwig A. Budde T. Stieber J. Moosmang S. Wahl C. Holthoff K.. (2003). Absence epilepsy and sinus dysrhythmia in mice lacking the pacemaker channel HCN2. EMBO J. 22, 216–224. 10.1093/emboj/cdg032 12514127 PMC140107

[B37] Ludwig A. Zong X. Jeglitsch M. Hofmann F. Biel M. (1998). A family of hyperpolarization-activated mammalian cation channels. Nature 393, 587–591. 10.1038/31255 9634236

[B38] Martin A. R. (1955). A further study of the statistical composition on the end-plate potential. J. Physiol. 130, 114–122. 10.1113/jphysiol.1955.sp005397 13278890 PMC1363457

[B39] Marx T. Gisselmann G. Störtkuhl K. F. Hovemann B. T. Hatt H. (1999). Molecular cloning of a putative voltage- and cyclic nucleotide-gated ion channel present in the antennae and eyes of *Drosophila melanogaster*. Invert. Neurosci. 4, 55–63. 10.1007/s101580050007 12491074

[B40] Pape H. C. (1996). Queer current and pacemaker: the hyperpolarization-activated cation current in neurons. Annu. Rev. Physiol. 58, 299–327. 10.1146/annurev.ph.58.030196.001503 8815797

[B41] Qu Y. Whitaker G. M. Hove-Madsen L. Tibbits G. F. Accili E. A. (2008). Hyperpolarization-activated cyclic nucleotide-modulated “HCN” channels confer regular and faster rhythmicity to beating mouse embryonic stem cells. J. Physiol. 586, 701–716. 10.1113/jphysiol.2007.144329 18033814 PMC2375615

[B42] Rieckhof G. E. Yoshihara M. Guan Z. Littleton J. T. (2003). Presynaptic N-type calcium channels regulate synaptic growth. J. Biol. Chem. 278, 41099–41108. 10.1074/jbc.M306417200 12896973

[B43] Robinson R. B. Siegelbaum S. A. (2003). Hyperpolarization-activated cation currents: from molecules to physiological function. Annu. Rev. Physiol. 65, 453–480. 10.1146/annurev.physiol.65.092101.142734 12471170

[B44] Santoro B. Liu D. T. Yao H. Bartsch D. Kandel E. R. Siegelbaum S. A.. (1998). Identification of a gene encoding a hyperpolarization-activated pacemaker channel of brain. Cell 93, 717–729. 10.1016/S0092-8674(00)81434-8 9630217

[B45] Shah M. M. (2014). Cortical HCN channels: function, trafficking and plasticity. J. Physiol. 592, 2711–2719. 10.1113/jphysiol.2013.270058 24756635 PMC4104471

[B46] Tomlinson S. Burke D. Hanna M. Koltzenburg M. Bostock H. (2010). *In vivo* assessment of HCN channel current (*I*_h_) in human motor axons. Muscle Nerve 41, 247–256. 10.1002/mus.21482 19813191

[B47] Trevillion L. Howells J. Bostock H. Burke D. (2010). Properties of low-threshold motor axons in the human median nerve. J. Physiol. 588, 2503–2515. 10.1113/jphysiol.2010.190884 20478975 PMC2915523

[B48] Wainger B. J. Degennaro M. Santoro B. Siegelbaum S. A. Tibbs G. R. (2001). Molecular mechanism of cAMP modulation of HCN pacemaker channels. Nature 411, 805–810. 10.1038/35081088 11459060

[B49] Weiszmann R. Hammonds A. S. Celniker S. E. (2009). Determination of gene expression patterns using high-throughput RNA in situ hybridization to whole-mount *Drosophila* embryos. Nat. Protoc. 4, 605–618. 10.1038/nprot.2009.55 19360017 PMC2780369

[B50] Xing B. Ashleigh Long A. Harrison D. A. Cooper R. L. (2005). Developmental consequences of neuromuscular junctions with reduced presynaptic calcium channel function. Synapse 57, 132–147. 10.1002/syn.20165 15945059

[B51] Zagotta W. N. Olivier N. B. Black K. D. Young E. C. Olson R. Gouaux E. (2003). Structural basis for modulation and agonist specificity of HCN pacemaker channels. Nature 425, 200–205. 10.1038/nature01922 12968185

