# Supplementary figures and images for: The Ih Channel Gene Promotes Synaptic Transmission and Coordinated Movement in Drosophila melanogaster
Source: Front Mol Neurosci. 2017 Feb 24;10:41. doi: 10.3389/fnmol.2017.00041 (PMC5323408; doi:10.3389/fnmol.2017.00041)

# Supplementary Figure 1

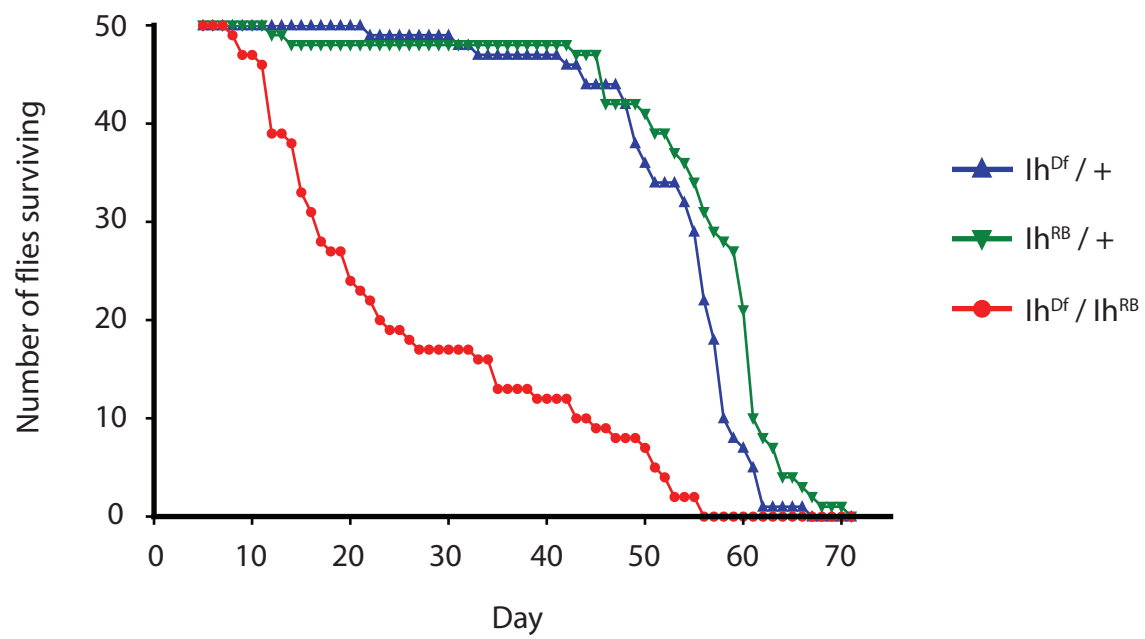

Supplement: Supplementary Figure 1 — Ih disruption reduces viability of Drosophila melanogaster adults. Graph showing the number of flies surviving over time for three genotypes. Flies with a P-element insertion in one copy (IhRB/+) have a normal lifespan of ~60 days, as do flies lacking the HCN locus on one chromosome (IhDf/+). In contrast, flies with disruptions in both copies of the HCN gene (IhDf/IhRB) have a significantly shortened lifespan (~20-day median). Starting populations of 50 flies per genotype were followed daily until all had died. [file Image1.PDF]
